# Movement Behaviors and Mental Wellbeing: A Cross-Sectional Isotemporal Substitution Analysis of Canadian Adolescents

**DOI:** 10.3389/fnbeh.2021.736587

**Published:** 2021-10-05

**Authors:** Denver M. Y. Brown, Matthew Y. W. Kwan

**Affiliations:** ^1^Department of Psychology, University of Texas at San Antonio, San Antonio, TX, United States; ^2^Department of Family Medicine, McMaster University, Hamilton, ON, Canada; ^3^Department of Child and Youth Studies, Brock University, St. Catharines, ON, Canada

**Keywords:** mental health, physical activity, screen time, sleep, daily time use

## Abstract

**Background:** Studies have shown reallocating screen time for healthy movement behaviors such as physical activity and sleep can provide important benefits for mental health. However, the focus on positive aspects of mental health such as wellbeing has received limited attention, particularly among adolescents. The purpose of this study was to examine the effects of reallocating physical activity, screen time, and sleep on mental wellbeing in adolescents.

**Methods:** This study involved cross-sectional analysis of data from Wave 1 of the ADAPT study. A total of 1,118 Canadian adolescents enrolled in grade 11 classes (M_AGE_ = 15.92; 54.5% female) self-reported their movement behaviors using the International Physical Activity Questionnaire – Short Form to assess moderate-to-vigorous physical activity and daily recall questionnaires to assess recreational screen time and sleep. Participants also completed three measures of mental wellbeing: the Flourishing Scale, Rosenberg Self-Esteem Scale, and a brief Resiliency scale from the Canadian Campus Wellbeing Survey.

**Results:** Isotemporal substitution analysis revealed replacing 60 min of screen time with either moderate-to-vigorous physical activity or sleep has significant benefits for mental wellbeing. Comparatively, reallocating 60 min between moderate-to-vigorous physical activity and sleep does not impact mental wellbeing.

**Discussion:** Findings suggest healthy movement behaviors confer similar beneficial effects for adolescent’s mental wellbeing. Health promotion campaigns targeted toward adolescents should consider highlighting that reallocation of screen time to either sleep or moderate-to-vigorous physical activity may provide important benefits for mental wellbeing.

## Introduction

The 24-h movement behavior paradigm is a relatively novel approach that considers how all types of movement behaviors – physical activity, sedentary behaviors, and sleep – collectively contribute to the healthy development of children and youth (Tremblay et al., 2016). This approach builds on previous work that typically examined independent associations between time spent in each movement behavior and health outcomes, and in doing so, accounts for the codependence among these behaviors over the course of a whole day (i.e., during the 24-h period) (Tremblay and Ross, 2020). Recent systematic reviews have shown engaging in a lifestyle consisting of adequate amounts of physical activity and sleep, in addition to a small amount of time spent engaging in sedentary behaviors such as recreational screen time has significant benefits for several important health indicators during the early life stages (Rollo et al., 2020; Sampasa-Kanyinga et al., 2020). Although research to date has largely focused on relationships between movement behaviors and physical health outcomes (e.g., adiposity, cardiometabolic health, aerobic fitness), indicators of mental health have begun to see increased attention of late, particularly among adolescents (Carson et al., 2016, 2017; Janssen et al., 2017; Knell et al., 2019; Pearson et al., 2019; Zhu et al., 2019; Bang et al., 2020; Faulkner et al., 2020; Patte et al., 2020; Brown et al., 2021a,b; Gilchrist et al., 2021; Sampasa-Kanyinga et al., 2021), yet critical knowledge gaps in our understanding of the movement behaviors – mental health relationship remain.

Understanding the impact of movement behaviors on mental health during the adolescent period is of particular importance from a public health standpoint as this life stage is characterized by heightened stress (Spear, 2000) and represents the peak onset of mental health problems (Kessler et al., 2007; Paus et al., 2008). Mental health, however, has been proposed to exist on a continuum that considers not only adverse symptoms, but also positive attributes (Keyes, 2002). From this perspective, it is equally as important that we establish empirical links between movement behaviors and mental wellbeing. Importantly, existing evidence suggests flourishing, self-esteem and resiliency – three indicators of mental wellbeing – may provide protective benefits against stress and the development of mental health problems such as depression and anxiety (Keyes, 2006; Orth et al., 2008; Hjemdal et al., 2011; Skrove et al., 2013; Kwok et al., 2014; Anyan and Hjemdal, 2016; Henriksen et al., 2017; Masselink et al., 2018; Doré et al., 2020).

To date, movement behaviors have been found to be associated with mental health and wellbeing (Sampasa-Kanyinga et al., 2020). However, the evidence has largely been based on adherence (or not) to each of the threshold-based 24-h movement guidelines (Carson et al., 2016, 2017; Janssen et al., 2017; Knell et al., 2019; Pearson et al., 2019; Zhu et al., 2019; Bang et al., 2020; Faulkner et al., 2020; Patte et al., 2020; Sampasa-Kanyinga et al., 2021), with findings generally indicating that concurrent adherence to all three threshold-based guidelines is associated with more favorable mental health and wellbeing than meeting two or fewer guidelines (Sampasa-Kanyinga et al., 2020). This approach is important from a behavioral surveillance standpoint in that it can provide population-level information about the proportion of adolescents meeting public health-based recommendations, but it is not without its limitations. For instance, using dichotomous cut-point criteria sacrifices a substantial amount of information about movement behaviors that may contribute to variability in indicators of mental health. This approach also fails to provide insight regarding what occurs when time spent in one movement behavior displaces another (e.g., staying up late to watch TV rather than sleep). Isotemporal substitution modeling is an alternative integrative approach that has been recommended to address these specific limitations (Chaput et al., 2017).

Isotemporal substitution modeling is particularly useful when considering 24-h movement guidelines, as time use across the course of a day is finite (i.e., cannot exceed 24-h). Therefore, engaging in one movement behavior comes at the cost of not engaging in other movement behaviors (Mekary et al., 2009). For example, if an adolescent wanted to go from being inactive to meeting the 24-h movement recommendation of 60 min of moderate-to-vigorous physical activity (MVPA) each day, then their combined time spent engaging in sedentary behaviors or sleep would be need reduced by 60 min. In addition to evaluating the reallocation of time spent in one movement behavior with another, the isotemporal substitution model also adjusts for the confounding effects of the remaining time use components (Mekary et al., 2009).

The authors of a recent systematic review of isotemporal substitution modeling of movement behaviors and related health outcomes highlighted that there is a dearth of studies related to mental health (Grgic et al., 2018). Evidence among adolescents is particularly limited, as only one study was found to have replaced time spent playing sedentary video games with active video games or active outside play was associated with positive influences on adolescent’s mental health (Janssen, 2016). However, this study failed to consider the full compliment of movement behaviors, as sleep was not assessed. Recently, there has been another study using cross-sectional data from over 46,000 students participating in the COMPASS Study. This study by Gilchrist and colleagues (2021) found that the benefits of reallocating 15 min of any type of behavior is moderated based on whether an adolescent meets the sleep guidelines. In other words, they found that replacing screen time with either homework, MVPA or sleep was generally associated with lower scores for anxiety and depression and higher scores for flourishing; however, the magnitudes of the effects for sleep were greatest when adolescents were not acquiring an average of 8 h of sleep each night. This seminal work established important empirical links between movement behaviors and flourishing, but additional research examining other indicators of mental wellbeing is needed to address key gaps in our current understanding. Therefore, the purpose of the current study was to examine the effects of reallocating physical activity, screen time, and sleep on indicators of mental wellbeing among a sample of Canadian adolescents. In line with previous research examining the relationship between movement behaviors and flourishing (Gilchrist et al., 2021), we hypothesized that replacing screen time with healthier movement behaviors (i.e., sleep and MVPA) would be associated with better mental wellbeing among adolescents getting less than the recommended amount of sleep, whereas among those getting enough sleep, replacing screen time and sleep with MVPA would result in better mental wellbeing.

## Materials and Methods

### Study Sample and Data Collection

The present study utilized baseline data from the ADAPT study: Application of integrated Approaches to understanding Physical activity during the Transition to emerging adulthood (Kwan et al., 2020). This is a 4-year, prospective longitudinal study tracking a sample of Canadian adolescents as they transition out of high school and into emerging adulthood in order to gain an understanding of factors underlying changes in physical activity behavior. At baseline, all grade 11 students enrolled into one of the seven secondary schools across a large school board in Southern Ontario were invited to take part in the study. Participation in the study was voluntary, and informed consent was obtained online from each participant prior to data collection at each individual school in the Fall/Autumn term. Students willing to participate completed a 20-min online survey during class time. Parental consent, collected by paper or electronically, was also a requirement for their data to be included in the study. More information about the recruitment strategy and study protocol can be found elsewhere (Kwan et al., 2020). The protocol for the ADAPT study was approved by both the Institutional Research Ethics Board and the School Board Ethics Committee.

Among the 2,412 enrolled at one of the seven secondary schools within the school board, 1,585 agreed and provided consent to participate (66% response rate). Of the 1,585 consenting students, 146 respondents (9%) withdrew their participation (i.e., completed >5% of the survey), while 186 respondents (12%) did not have parental consent for their participation. As a result, the final baseline sample in the current study included 1,253 participants. Among the total baseline cohort, 1,165 participants (93%) had full data for the variables of interest and covariates used in our analyses. Missing data included 16 cases for movement behaviors (1%), 34 (3%) for at least one mental wellbeing variable, 26 (2%) for gender, and 12 (1%) for parental education. Missing data was handled using listwise deletion. Additionally, 47 participants reported amounts of physical activity, recreational screen time and sleep that totaled over 24 h, and were therefore removed from the present analysis, resulting in data for a total of 1,118 participants included in this study.

### Measures

#### Demographics

Participants completed a demographic questionnaire assessing their age, gender, ethnicity, and highest level of parental education. For analyses purposes, ethnicity was dummy coded into White (1) and other (0), and parental education was dummy coded into college/university graduate (1) and partial completion of post-secondary education or less (0). Parental education was used as a proxy indicator of socioeconomic status.

#### Movement Behaviors

In accordance with the Canadian 24-Hour Movement Guidelines for Children and Youth (1), movement behaviors were operationalized as a latent construct consisting of MVPA, screen time and sleep. The present study only focused on the three threshold-based components of the 24-h cycle despite recommendations for engaging in several hours of light physical activity also included within the guidelines.

##### Moderate-to-Vigorous Physical Activity

Moderate-to-vigorous physical activity was measured using the International Physical Activity Questionnaire – Short Form (Booth, 2000; Craig et al., 2003). Participants responded to four items that assessed the frequency (days) and duration (hours and/or minutes on an average day) of their moderate and vigorous physical activity performed in bouts of greater than 10-min over the past 7 days. The International Physical Activity Questionnaire – Short Form defines moderate physical activity as “activities that take moderate physical effort and make you breathe somewhat harder than normal” and provides carrying light loads, bicycling at a regular pace, and playing doubles tennis, but not walking, as examples of activities that involve moderate physical effort. As per the International Physical Activity Questionnaire – Short Form, vigorous physical activity is defined as “activities that take hard physical effort and make you breathe much harder than normal,” with heavy lifting, digging, aerobics, or fast bicycling provided as examples. Daily MVPA was calculated by multiplying frequency by duration for moderate and vigorous physical activity, respectively, and then summing these products and dividing by seven. The International Physical Activity Questionnaire – Short Form has shown acceptable measurement properties when administered among adolescents (Guedes et al., 2005).

##### Screen Time

Screen time was assessed using a standard daily recall questionnaire that asked participants how much time (hours and/or minutes) on average they spent watching TV or using a computer, tablet or smartphone during their free time over the past 7 days.

##### Sleep

Participants responded to four items that assessed what time they typically went to sleep and woke up during weekdays and on the weekend over the past 7 days. Responses were used to calculate the average number of hours participants slept on weekdays and weekends. Average daily sleep was then calculated by multiplying weekday sleep by five and weekend sleep by two, and then summing these products and dividing by seven. Although sleep quality may provide additional insight that could be used to understand the relationship between sleep and mental health (João et al., 2018), recommendations provided in the 24-Hour Movement Guidelines for Children and Youth focus on sleep duration – 8 to 10 h for youth – for healthy development.

#### Flourishing

Flourishing was measured using the Flourishing Scale (Diener et al., 2010), which provides a summary measure of the respondent’s self-perceived success in important aspects of their life including relationships, purpose and optimism. This measure has demonstrated strong psychometric properties when administered to youth (Diener et al., 2010). Participants responded to eight items on a seven-point Likert scale ranging from 1 (Strongly Disagree) to 7 (Strongly Agree). Example items included: “I lead a purposeful and meaningful life,” “My social relationships are supportive and rewarding,” and “I am optimistic about my future.” All items were summed to provide a total score ranging from 8 to 56. Internal consistency (Cronbach’s α) for the eight items was 0.89.

#### Self-Esteem

Self-esteem was measured using a modified version of the Rosenberg Self-Esteem Scale (Rosenberg, 1965). This measure has demonstrated strong psychometric properties when administered to adolescents (Rosenberg, 1965). Participants responded to five items on a four-point scale ranging from 1 (Strongly Disagree) to 4 (Strongly Agree). Only the items associated with positive feelings toward the self were included: “On the whole, I am satisfied with myself,” “I feel that I have a number of good qualities,” “I am able to do things as well as most other people,” “I feel that I’m a person of worth, at least on an equal plane with others,” and “I take a positive attitude toward myself.” All items were summed to provide an overall score ranging from 5 to 20. Internal consistency (Cronbach’s α) for the five items was 0.88.

#### Resiliency

Resiliency was measured using two items from the Canadian Campus Wellbeing Survey (Faulkner et al., 2019). Participants responded to each item on a five-point scale ranging from 1 (Poor) to 5 (Excellent). The items followed the stem statement: “In general, how would you rate…”: (a) “your ability to handle unexpected and difficult problems (a family or performance crisis),” and (b) “your ability to handle day-to-day demands in your life (work, family responsibilities).” A mean scale score was computed. The inter-item correlation (Pearson’s *r*) for the two items was 0.72.

### Data Analysis

Descriptive statistics and frequencies were computed for each variable and distributions were examined for normality. Participants with missing data for the variables of interest and covariates were removed, as were cases with unrealistic responses (i.e., average daily time use spent in movement behaviors exceeding 24 h). Isotemporal substitution modeling requires an approximate linear association between predictor variables and outcomes; and therefore, we examined whether significant quadratic relationships existed given that previous work has found non-linear associations between sleep and indicators of mental health (Gilchrist et al., 2021). Our primary analysis consisted of using two different regression models to examine relationships between movement behaviors and indicators of mental wellbeing: (1) single activity, and (2) isotemporal substitution.

First, we computed a series of single activity models to investigate independent associations between each movement behavior (i.e., MVPA, screen time, sleep) and the three indicators of mental wellbeing. Single activity models were expressed as follows:

Mental⁢wellbeing=(β⁢1)⁢MVPA+(β⁢2)⁢covariates.

Next, a series of isotemporal substitution models were computed to examine the effects of reallocating time between MVPA, screen time and sleep on indicators of mental wellbeing. Isotemporal substitution models estimate the effects of replacing time spent engaging in one behavior with another behavior for the same amount of time (Mekary et al., 2009). By replacing a reduction in time spent engaging in one behavior with an equivalent amount of time in another behavior, total time is held constant in the equation, and the potential to create a day longer than 24 h is eliminated. Given that individuals generally spend the least amount of their day engaging in MVPA, we modeled the impact of replacing 60 min of time use in one movement behavior with another activity to represent the effects of going from being inactive (i.e., 0 min of daily MVPA) to meeting the guideline recommendation of 60 min of MVPA per day, and vice versa. Using the reallocation of MVPA to screen time or sleep as an example, the isotemporal substitution models were expressed as follows, in which the coefficients β1 and β2 express the revised estimate for mental wellbeing associated with a 60 min increase in the values for screen time and sleep at the expense of MVPA, respectively, whereas the coefficient β3 (total time), which holds time constant in the equation, represents the revised estimate for mental wellbeing associated with the activity that was replaced (i.e., MVPA):

Mental⁢wellbeing=(β⁢1)⁢screen⁢time+(β⁢2)⁢sleep+

(β⁢3)⁢total⁢time+(β⁢4)⁢covariates

Multilevel modeling was used to account for the nested structure of the data (i.e., participants within schools). Prior to conducting our analyses, all relevant assumptions (i.e., linearity, homogeneity, normality) were tested. Assumptions for linearity and homogeneity were met, however, the normal probability plots of residuals had considerable outliers and were not normally distributed. Mahalanobis tests indicated there were 68, 67, and 54 multivariate outliers for flourishing, self-esteem and resiliency, respectively. Since ordinary least squares regression estimates are sensitive to outliers and highly influential observations, robust regression was employed to reduce the influence of these observations. This approach decreases the weights of observations with large residuals to reduce their influence on model estimates. For our robust estimates, we used a smoothed Huber function with the tuning parameter set at *k* = 1.345 and *s* = 10 to ensure our models achieved 95% efficiency relative to the ordinary least squares estimates (Koller, 2016). Each model included gender, socioeconomic status, and race/ethnicity as covariates. Parameter estimates (β) and standard errors (SE) are reported for the single activity, and isotemporal substitution models. All analyses were performed in R (Version 4.0.3) and R Studio (Version 1.3.1093) using the *psych* (Revelle, 2011), *lme4* (Bates et al., 2015), and *robustlmm* packages (Koller, 2016). Example R-code for the analysis can be found in the Supplementary Material. Statistical significance was set at *p* < 0.01, which, for robust estimates, corresponds with *z*-values ≥ 2.58.

## Results

Descriptive statistics for the sample are presented in Table 1. The sample was on average 16 years of age and comprised of slightly more females than males. The majority of participants identified as White, and most participants lived in a household with a parent who graduated from college or university. With the exception of the relationship between sleep and flourishing, we failed to observe evidence of significant quadratic relationships between each movement behavior and the indicators of mental wellbeing. To assess the non-linear association between sleep and flourishing, we stratified our analyses for flourishing based on whether participants met the lower bound age-associated sleep threshold recommendation for youth (≥8 h of sleep each night; *n* = 284) or not (<8 h of sleep each night; *n* = 834). (1) Analyses for self-esteem and resiliency are non-stratified.

**TABLE 1:** Demographic characteristics (*N* = 1,118).

	Total sample
	*n* (%) or *M* (SD)
Age	15.92 (0.49)
Gender (male)	509 (45.5)
**Race/Ethnicity**	
White	589 (52.7)
Indigenous	22 (2.0)
Black	91 (8.1)
Asian	66 (5.9)
Middle Eastern/Arab	105 (9.3)
Latin	4.5 (50)
Other	195 (17.4)
**Highest parental education**	
Some secondary	139 (12.4)
Completed secondary	85 (7.6)
Some post-secondary	118 (10.6)
Completed post-secondary	776 (69.4)
Moderate-to-vigorous physical activity (hours/day)	1.36 (0.98)
Screen time (hours/day)	4.68 (3.17)
Sleep duration (hours/day)	7.29 (1.40)

### Single and Isotemporal Substitution Models

Two different models were computed to examine associations between time use in different movement behaviors (including reallocation) and three indicators of mental wellbeing among adolescents (Table 2).

**TABLE 2:** Single and isotemporal substitution models predicting indicators of mental wellbeing.

	Replacement activity		
	MVPA	Sleep	Screen
	B (SE)	B (SE)	B (SE)
*Flourishing (*≥*8 h of sleep)*			
Isotemporal substitution			
MVPA	–	**−1.64 (0.59)**	**−1.22 (0.45)**
Sleep	**1.65 (0.59)**	–	0.42 (0.38)
Screen time	**1.23 (0.45)**	−0.42 (0.38)	–
Single activity	0.90 (0.41)	−0.54 (0.34)	−0.15 (0.15)
*Flourishing (*<*8 h of sleep)*			
Isotemporal substitution			
MVPA	–	0.05 (0.37)	**−2.02 (0.27)**
Sleep	−0.05 (0.37)	–	**−2.07 (0.26)**
Screen time	**2.02 (0.27)**	**2.07 (0.26)**	–
Single activity	**1.95 (0.28)**	**2.03 (0.26)**	**−0.35 (0.08)**
*Self-esteem*			
Isotemporal substitution			
MVPA	–	0.05 (0.11)	**−0.48 (0.09)**
Sleep	−0.05 (0.11)	–	**−0.53 (0.06)**
Screen time	**0.48 (0.09)**	**0.53 (0.06)**	–
Single activity	**0.45 (0.09)**	**0.49 (0.06)**	**−0.10 (0.03)**
*Resiliency*			
Isotemporal substitution			
MVPA	–	−0.08 (0.03)	**−0.16 (0.03)**
Sleep	0.08 (0.03)	–	**−0.08 (0.02)**
Screen	**0.16 (0.03)**	**0.08 (0.02)**	–
Single activity	**0.16 (0.03)**	**0.08 (0.02)**	**−0.02 (0.01)**

*Estimates are unstandardized. SE, standard error. All models are adjusted for sex, race/ethnicity, and socioeconomic status. Significant findings are bolded (*p* < 0.01).*

#### Single Activity Models

Sleep duration and MVPA were positively associated with both self-esteem and resiliency, whereas a negative association was observed for screen time. Similar results were also found for flourishing among the subsample of participants getting less than 8 h of sleep. Comparatively, no significant associations were observed between each movement behavior and flourishing for the subsample getting at least 8 h of sleep.

#### Isotemporal Substitution Models

Reallocating 60 min of daily screen time to MVPA or sleep was associated with significantly better self-esteem and resiliency. Alternatively, there were no changes in self-esteem and resiliency when sleep was replaced with MVPA (and vice versa). For flourishing, significant benefits were observed when replacing screen time with MVPA. When screen time was replaced with sleep, the beneficial effects on flourishing were only observed for the subsample averaging less than 8 h of sleep. When sleep was reallocated to MVPA, no differences were observed for flourishing among the subsample averaging less than 8 h of sleep, but resulted in significantly better flourishing scores among the subsample averaging at least 8 h of sleep each night. Interestingly, for adolescents averaging more than 8 h of sleep per night, the reallocation of time spent sleeping to screen time had no effect on flourishing.

## Discussion

The present study examined the impact of reallocating time spent engaging in different movement behaviors on indicators of mental wellbeing among a sample of Canadian adolescents. These results generally suggest that replacing 60 min of screen time with either sleep or MVPA confers beneficial effects for flourishing, self-esteem and resiliency. Replacing sleep with MVPA (and vice versa), however, appears to have a negligible effect on self-esteem and resiliency. Getting additional MVPA at the expense of sleep or screen time appears to provide the greatest benefits for flourishing among adolescents getting sufficient sleep, whereas replacing screen time with MVPA or sleep is associated with similar benefits among adolescents achieving insufficient amounts of sleep. Collectively, findings have important public health implications, particularly regarding how health promotion campaigns frame the detrimental impact for mental wellbeing of spending time using screens at the expense of being active or resting.

Emerging research is beginning to establish how varying combinations of daily MVPA, sleep and sedentary behaviors interact to influence the mental health and wellbeing of adolescents (Sampasa-Kanyinga et al., 2020). The present study builds on the limited literature, demonstrating that replacing screen time with either MVPA or sleep is associated with higher levels of not only flourishing (Gilchrist et al., 2021), but also other important indicators of mental wellbeing that have been shown to confer protective effects against stress and the development of mental health problems. These findings are important considering recent work using a rigorous multiverse analysis has suggested that screen time alone may not be worth targeting from a policy level standpoint when attempting to improve the mental wellbeing of adolescents (Orben and Przybylski, 2019). In light of the 24-h movement paradigm, however, the true benefits may lie in the replacement of screen time with healthier alternatives (i.e., MVPA, sleep) that are known to have a positive impact on mental wellbeing. Our findings, therefore, underscore the need for intervention efforts aiming to relocate some time away from sedentary pursuits to increase the proportion of an adolescent’s day that is allocated to time spent engaging in MVPA or sleep. Evidence indicates that a health promotion campaign focused on getting adolescents to turn off their screens earlier in the evening and get to sleep at a more reasonable time may hold promise for promoting their mental wellbeing (Woods and Scott, 2016).

Although we observed a quadratic relationship between sleep and flourishing that was consistent with findings of Gilchrist et al. (2021), self-esteem and resiliency both displayed linear relationships with sleep and were therefore not stratified based on sleep duration as per our hypotheses. The results for both of these indicators of mental wellbeing would suggest that replacing screen time with either sleep or MVPA confers beneficial effects of similar magnitudes. Considering the average amount of time we found adolescents spend using screens on a daily basis was more than double the recommended 2 h threshold and roughly three-quarters of the sample averaged less than 8 h of sleep each night, there appears to be ample opportunity to enhance wellbeing through replacement of time on screens with sleep later in the evening. Moreover, the relative equivalence in beta coefficients for replacing screen time with MVPA and sleep means that adolescents that potentially find MVPA aversive can still stand to improve their wellbeing simply through increasing their sleep duration. However, while focusing on replacing screen time with sleep may lead to improvements in mental wellbeing, failing to take an integrative approach that also considers MVPA could come at the expense of improvements in indices of physical health such as adiposity and cardiometabolic biomarkers (Grgic et al., 2018; Janssen et al., 2020). As more research emerges, we will gain a better understanding of how time spent amongst different movement behaviors can be best allocated to optimize a broad range of health outcomes, collectively.

Consistent with our hypotheses based on previous research (Gilchrist et al., 2021), the relationship between sleep and flourishing was best characterized as quadratic rather than linear, which led us to stratify our analyses for flourishing based on whether participants met the lower bound of the 24-h movement guideline recommendations for sleep [i.e., averaging 8 h of sleep each night (1)]. Our results for flourishing align with Gilchrist et al. (2021) in that the direction and magnitude of the observed associations for reallocating time amongst movement behaviors were predicated based on sleep duration. For the subsample of participants getting less than 8 h of sleep each night – which represents ∼75% of the sample – the pattern of results was equivalent to what was found for self-esteem and resiliency. In contrast, when adolescents met the sleep guidelines, they can stand to engage in more screen time at the expense of sleep without sacrificing their mental wellbeing, and replacing sleep with MVPA can even lead to beneficial effects for flourishing. Although replacing screen time with MVPA was positively associated with flourishing for both groups, it is worth noting that the magnitude of the effect was greater for the group not meeting current sleep guidelines. These results ultimately suggest that flourishing is more stable in adolescents who meet the sleep guidelines, and thus may provide a stronger buffer against time spent using screens instead of being active.

As depression and other mental health problems become a larger public health concern (World Health Organization [WHO], 2017), it is imperative that we identify low-cost strategies to combat these issues when they begin to manifest. Our findings indicate that health promotion strategies targeting the replacement of recreational screen time with healthy alternatives such as MVPA and sleep can benefit adolescent’s mental wellbeing. Such efforts may in turn buffer against the high levels of stress and onset of mental health problems experienced during this key developmental stage. These results provide further support for the global shift toward adopting a multi-faceted approach to healthy development through targeting MVPA, sedentary behaviors and sleep concurrently. Health promotion campaigns and behavior change interventions targeted and tailored to subgroups engaging in suboptimal movement behavior patterns could represent effective means for improving population mental health from a young age (Fisher, 2021). In fact, two recent studies employing latent profile analysis have shown that adolescents classified into groups characterized by the least healthy combination of movement behaviors (i.e., low MVPA, high screen time) report the poorest scores across several indicators of mental health and wellbeing (Brown et al., 2021a,b). Evidently, this is an ideal subsample of the adolescent population to target with interventions focused on reallocating screen time to other movement behaviors. Optimal strategies by which this can be accomplished are currently unknown, but future research in this area is clearly warranted.

There are, however, limitations to this study that should be noted. First, this study was cross-sectional and only used data from the first wave of the ADAPT study, and therefore causal links cannot be inferred. As future waves of data are collected from the ADAPT study cohort, we will be able to use longitudinal modeling to establish causal links. Researchers with access to longitudinal datasets that include measures of movement behaviors and mental wellbeing are encouraged to begin exploring these links. Second, movement behaviors were assessed using self-reported measures which can introduce response bias. Third, the measures used to assess MVPA and screen time do not provide domain-specific information which may have differential effects on adolescent wellbeing. For example, meta-analytic evidence has established significant positive associations between leisure-time physical activity and wellbeing, whereas the associations with other domains of physical activity (e.g., school sport, physical education, occupational) are less conclusive (White et al., 2017). Examining specific types of physical activity and screen time in future studies may provide important information to guide precision medicine interventions. A fourth limitation relates to treating time use spent engaging in movement behaviors as absolute (i.e., hours/day) as opposed to relative (i.e., proportion of the 24-h window). Some researchers have suggested that isotemporal substitution modeling of time use data is best suited for compositional data analysis techniques given that time spent engaging in different behaviors is codependent and bounded to the 24-h period (Dumuid et al., 2019), whereas others have argued that the traditional method using absolute values provides equivalent results that may even be easier to understand given that the 24-h movement guidelines provide absolute rather than relative time-based recommendations (Mekary and Ding, 2019). Regardless of the isotemporal substitution method employed, a shortcoming of this technique is that more time can be reallocated away from a behavior than the amount reported/measured due to the hypothetical nature of these models, and thus, precision of the estimates may be biased. Nevertheless, the traditional isotemporal substitution model was considered to be more appropriate in this case as time spent engaging in MVPA and screen time may not be codependent (e.g., using the treadmill while watching TV) as would be expected when using accelerometry which captures movement behaviors purely based on intensity of motion (i.e., sleep, sedentary behavior, light physical activity, MVPA).

In conclusion, we found that substituting an hour of daily time use spent using screens with MVPA or sleep can lead to improvements in adolescent mental wellbeing. While the reallocations of MVPA with sleep (and vice versa) generally had limited impact, adolescents who meet the sleep guidelines may experience additional benefits for their self-perceived success by sacrificing an hour of sleep for MVPA. Together, findings from this research highlight the need for future health promotion campaigns and interventions adopting the 24-h movement paradigm to focus on reallocating screen time use to healthier pursuits that adolescents enjoy engaging in most.

## Data Availability Statement

The raw data supporting the conclusions of this article will be made available by the authors, without undue reservation.

## Ethics Statement

This study involved human participants and was reviewed and approved by the McMaster Research Ethics Board and the Hamilton-Wentworth Catholic District School Board Ethics Committee. Written informed consent to participate in this study was provided by the participants’ legal guardian/next of kin.

## Author Contributions

DB: conceptualization, data curation, methodology, writing – original draft, and formal analysis. MK: writing – original draft, supervision, funding acquisition, and investigation. Both authors contributed to the article and approved the submitted version.

## Conflict of Interest

The authors declare that the research was conducted in the absence of any commercial or financial relationships that could be construed as a potential conflict of interest.

## Publisher’s Note

All claims expressed in this article are solely those of the authors and do not necessarily represent those of their affiliated organizations, or those of the publisher, the editors and the reviewers. Any product that may be evaluated in this article, or claim that may be made by its manufacturer, is not guaranteed or endorsed by the publisher.
