# Supplementary material for: Movement Behaviors and Mental Wellbeing: A Cross-Sectional Isotemporal Substitution Analysis of Canadian Adolescents
Source: Front Behav Neurosci. 2021 Oct 5;15:736587. doi: 10.3389/fnbeh.2021.736587 (PMC8523680; doi:10.3389/fnbeh.2021.736587)
Supplement: Supplementary file 1 [file Table_1.DOCX]

**Supplementary File:** R code for isotemporal substitution modeling for Brown & Kwan - Movement behaviours and mental wellbeing: A cross-sectional isotemporal substitution analysis of Canadian adolescents.

#read data in to RStudio

adapt <- read.csv("adapt.csv")

#specify which variables are factors

adapt$school <- as.factor(adapt$school)

adapt$sex <- as.factor(adapt$sex)

adapt$ses <- as.factor(adapt$ses)

adapt$race <- as.factor(adapt$race)

# REMOVE UNREALISTIC TIME USE CASES

# remove all cases in which moderate-to-vigorous physical activity (MVPA), screen time and
# sleep sum up to more than 24 hours

adapt <- subset(adapt, total < 24.0001)

# COMPARE LINEAR VS. QUADRATIC RELATIONSHIPS

# determine whether independent associations between each movement behaviour and each
# indicator of mental wellbeing are better characterized as linear or quadratic

# for brevity sake, only showing code for MVPA and flourishing

library("lme4")

# quadratic model

MVPA_flour_quad <- lmer(adapt$flour ~ adapt$mvpa + I(adapt$mvpa^2) + (1|adapt$school), REML = FALSE)

summary(MVPA_flour_quad)

# linear model

MVPA_flour <- lmer(adapt$flour ~ adapt$mvpa + (1|adapt$school), REML = FALSE)

summary(MVPA_flour)

# compare models

anova(MVPA_flour_quad,MVPA_flour)

# except for the relationship between sleep and flourishing, no significant quadratic relationships
# were observed, and therefore, all self-esteem and resiliency models were computed with the
# full sample

# for flourishing, however, all of the following analyses were computed with participants split
# into two groups based on whether they engaged in at least 8 hours of sleep on average each
# night or not, in accordance with the lower bound threshold of the youth sleep recommendations # from the 24-h movement guidelines (Tremblay et al., 2016)

# FLOURISHING ONLY- split based on average sleep duration

adapt0 <- adapt[adapt$sleep < 8,]

adapt1 <- adapt[adapt$sleep >= 8,]

# IDENTIFY MULTIVARIATE OUTLIERS

library("psych")

# example using self-esteem

OLmbe <-cbind(adapt$mvpa, adapt$screen, adapt$sleep, adapt$esteem)

outlier(OLmbe) # q-q plot

OLmbe.center = colMeans(OLmbe)

OLmbe.cov = cov(OLmbe)

rad = qchisq(p = 0.975 , df = ncol(OLmbe))

rad = sqrt(rad)

distances <- mahalanobis(x = OLmbe , center = OLmbe.center , cov = OLmbe.cov)

cutoff <- qchisq(p = 0.975 , df = ncol(OLmbe))

OLmbe[distances > cutoff ,]

names_outliers_mbe <- which(distances > cutoff)

names_outliers_mbe # show which cases were classified as outliers and removed

# for single, partition, and isotemporal substitution models, load robustlmm package to handle
# non-normal distribution of residuals and the considerable amount of multivariate outliers
# observed

library("robustlmm")

# SINGLE ASSOCIATION MODELS

# for brevity’s sake, only showing code for MVPA and self-esteem model

MVPA_esteem <- rlmer(adapt$esteem ~ adapt$mvpa + adapt$sex + adapt$race + adapt$ses + (1|adapt$school))

summary(MVPA_esteem)

# ISOTEMPORAL SUBSTITUTION MODELS

# for brevity’s sake, only showing code for self-esteem model with sleep replaced

esteem_isoSLEEP_ROB <- rlmer(adapt$esteem ~ adapt$mvpa +adapt$screen + adapt$total + adapt$sex + adapt$race +adapt$ses + (1|adapt$school))

summary(esteem_isoSLEEP_ROB)

plot(esteem_isoSLEEP_ROB)
